# Starting a Prehospital Medication for Opioid Use Disorder Program

**DOI:** 10.1017/S1049023X24000475

**Published:** 2024-10

**Authors:** David C. Seaberg, Jamie McKinnon, Lyn Haselton, Doug Gallo, Jason Kolb, Mary Moran, Suman Vellanki, Amy Raubenolt, Erin Simon, Nicholas Jouriles

**Affiliations:** 1. Department of Emergency Medicine, Summa Health System, Northeast Ohio Medical University, Akron, Ohio USA; 2. Department of Psychiatry, Summa Health System, Northeast Ohio Medical University, Akron, Ohio USA; 3. Department of Emergency Medicine, Cleveland Clinic – Akron General Hospital, Cleveland, Ohio USA

**Keywords:** emergency medical systems, medication for opioid use disorder, opioid addiction

## Abstract

**Background:**

Over 2.7 million people have an opioid use disorder (OUD). Opioid-related deaths have steadily increased over the last decade. Although emergency department (ED)-based medication for OUD (MOUD) has been successful in initiating treatment for patients, there still is a need for improved access. This study describes the development of a prehospital MOUD program.

**Methods:**

An interdisciplinary team expanded a MOUD program into the prehospital setting through the local city fire department Quick Response Team (QRT) to identify patients appropriate for MOUD treatment. The QRT consisted of a paramedic, social worker, and police officer. This team visited eligible patients (i.e., history of an opioid overdose and received prehospital care the previous week). The implementation team developed a prehospital MOUD protocol and a two-hour training course for QRT personnel. Implementation also required a signed contract between local hospitals and the fire department. A drug license was necessary for the QRT vehicle to carry buprenorphine/naloxone, and a process to restock the vehicle was created. Pamphlets were created to provide to patients. A clinical algorithm was created for substance use disorder (SUD) care coordinators to provide a transition of care for patients. Metrics to evaluate the program included the number of patients seen, the number enrolled in an MOUD program, and the number of naloxone kits dispensed. Data were entered into iPads designated for the QRT and uploaded into the Research Electronic Data Capture (REDCap) program.

**Results:**

Over the six-month pilot, the QRT made 348 visits. Of these, the QRT successfully contacted 83 individuals, and no individuals elected to be evaluated for new MOUD treatment. Nine fatal opioid overdoses occurred during the study period. A total of 55 naloxone kits were distributed, and all patients received MOUD information pamphlets.

**Conclusions:**

A prehospital MOUD program can be established to expand access to early treatment and continuity of care for patients with OUD. The program was well-received by the local city fire department and QRT. There is a plan to expand the prehospital MOUD program to other local fire departments with QRTs.

## Introduction

The United States has seen a significant increase in prescription drug misuse, leading to the opioid crisis. In 2020, an estimated 2.7 million people in the United States had an opioid use disorder (OUD).^1^ The annual OUD-related cost to the United States was US$786.8 billion in 2018.^2^

Despite the attention OUD has received, opioid deaths have significantly increased over the last decade.^3^ The emergence of emergency department (ED)-based medication for OUD (MOUD) programs has been beneficial in expanding access to treatment.^4,5^ However, there are individuals in the community who are struggling, but for reasons that have not been firmly identified, are simply not connecting with addiction treatment services. Recent reports describing escalating rates of substance abuse^2,3^ related to the pandemic highlight the importance of continued programmatic growth to ensure sufficient access to potentially life-saving treatment. However, in the Akron, Ohio area, Emergency Medical Services (EMS) are observing an ever-increasing number of patients who, due to refusing transport after naloxone rescue, represent an access void at the point of overdose. Prehospital initiation of buprenorphine treatment for OUD by paramedics is an emerging potential intervention.^6,7^ Many patients who may be at high risk for overdose deaths may never engage in treatment because they frequently refuse transport. Recent data have demonstrated a significant increase in both short- and long-term mortality following an opioid overdose.^8^

To address these ongoing challenges, the primary purpose of this study was the development of an interdisciplinary program aimed at expanding access to MOUD through a prehospital setting.

## Methods

The interdisciplinary team expanded two hospital based MOUD programs into the prehospital setting through the Akron Fire Department (AFD; Akron, Ohio, USA). The team worked with AFD to identify appropriate patients in real-time for treatment and referral of OUD patients. In 2022, AFD had 45,216 encounters. Of the total calls in 2022, 2.5% were for overdoses. They have several innovative programs that work to engage the community, identify patients for OUD treatment, and reduce EMS and ED visit utilization in this patient population.

The team received an Ohio Department of Health (Columbus, Ohio, USA) grant, which allowed AFD and the two major health systems in Akron that provide MOUD to collaborate. Both hospitals provide addiction care coordinators (ACCs) and peer counselors to help engage patients in their MOUD program. The Summa Health Institutional Review Board approved the program as an exempt quality improvement initiative.

**Figure 1.**
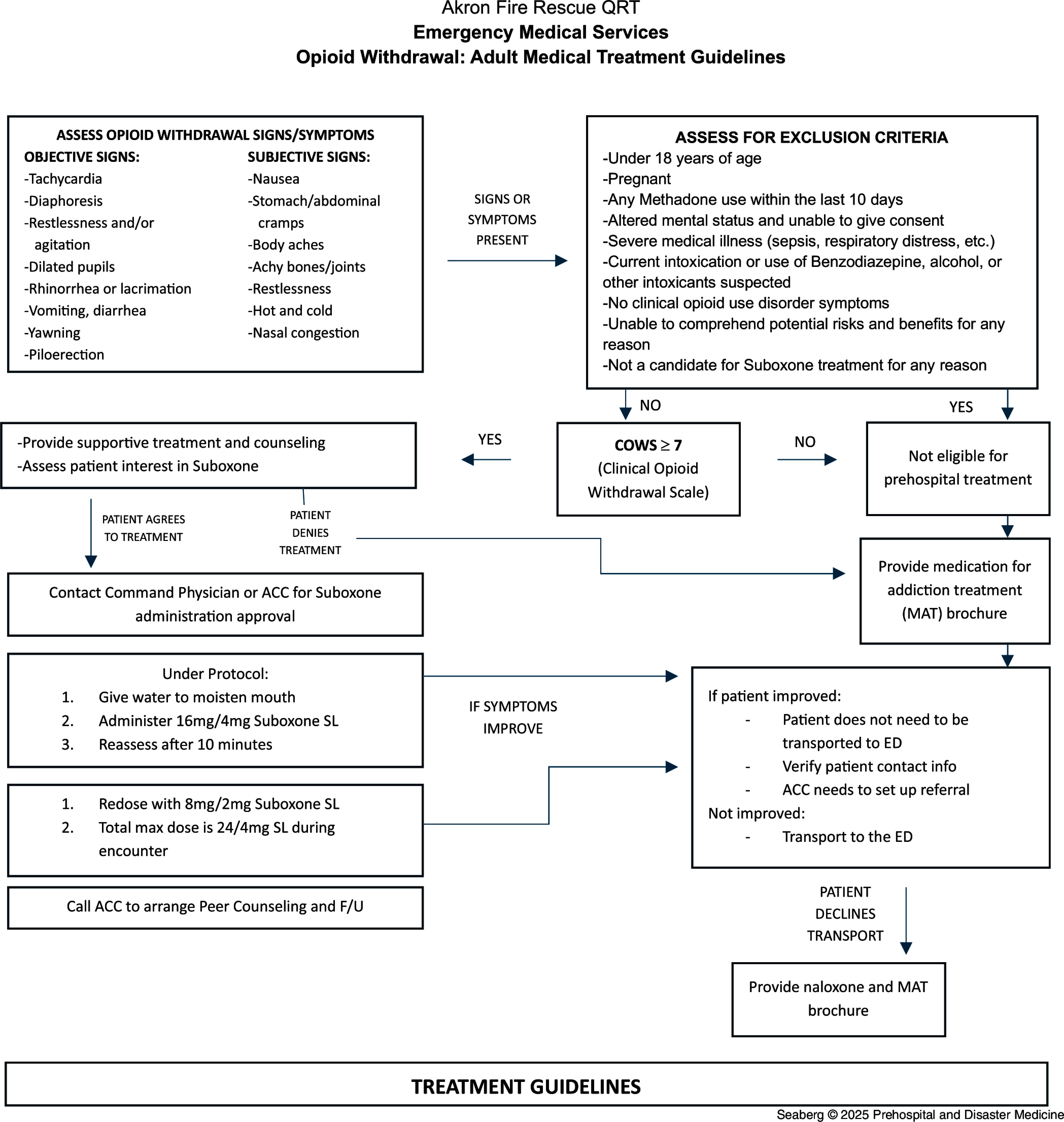
Akron Fire Department Prehospital MOUD Protocol. Abbreviations: ACC, addiction care coordinator; COWS, clinical opiate withdrawal scale; ED, emergency department; F/U, follow-up; MAT, medication for addiction treatment; MOUD, medication for opioid use disorder; QRT, Quick Response Team.

The AFD has a Quick Response Team (QRT) that visits people struggling with OUD to assist in guiding them toward definitive care. The program includes a team comprised of a paramedic, police officer, and Recovery Coach employed by the Summit County Health Department (Akron, Ohio, USA). This team visited eligible patients (i.e., history of an opioid overdose and received prehospital care the previous week and did not have an active warrant for arrest) at the patient’s home every Thursday. To ensure the program’s success, the implementation team developed a prehospital MAT protocol (Figure 1) and a two-hour training course for QRT personnel. Consultation with an ACC and peer counseling was offered to each patient. Each week, AFD had between three to eight opioid overdose patients who received naloxone and were either transported to the hospital or refused transport.

The implementation steps included six separate components. First, the development of a contract between AFD and the two area hospitals to provide prehospital MOUD was required. Second, QRT protocols were revised to incorporate MOUD. Third, a two-hour mandatory MAT training for the QRT was developed, which included an overview of the MAT program, the pharmacology of MAT programs, a treatment protocol review, patient follow-up education, and data collection through the Research Electronic Data Capture (REDCap) program (Vanderbilt University; Nashville, Tennessee USA). Fourth, a process for telemedicine consults with a hospital-based ACC using iPads (Apple Inc.; Cupertino, California, USA) was developed. Fifth, an application was made to the state for a drug license for the QRT vehicle to carry suboxone. A policy was created for hospital pharmacies to stock and restock suboxone on the QRT vehicle. Lastly, each hospital developed a pamphlet describing their MOUD programs and how to access addiction services if they refused prehospital MOUD.

The effectiveness of the QRT team in enrolling OUD patients in the prehospital MOUD program was evaluated. Outcome metrics included the number of patients seen by the QRT, the number of patients who were enrolled in the prehospital MOUD program, and the number of pamphlets and naloxone kits dispensed. A REDCap electronic survey was developed to collect data on individuals being visited. During the visit, the REDCap survey was used to collect data related to the treatment options that the patient accepted by the QRT member: (1) Patient elected to participate in the treatment program (e.g., spoke to an ACC or was transported to an ED to speak to one); (2) Patient had an ACC virtual visit; (3) Patient was transported to an ED; (4) Patient was administered suboxone; (5) Patient was given a naloxone kit; and (6) Patient connected to a -peer recovery specialist.

## Results

The project team worked with AFD to identify appropriate patients in real time for treatment and referral of OUD. The QRT made 348 visits during the six-month study period. Of these, QRT successfully contacted 83 individuals, and no individuals elected to be evaluated for MAT treatment or survey participation. Nine fatal opioid overdoses occurred during the study period.

The QRT visits successfully facilitated the delivery of life-saving naloxone kits in 55 (66%) patients and the dissemination of MOUD program education, resources, and Hope bags (a small bag with first aid supplies and a list of community resources) to all 83 patients.

## Discussion

This study aimed to increase access and patient enrolment to MOUD in a prehospital setting through an interdisciplinary team. However, no individuals elected to participate in the program. Possible contributing factors may have included patient readiness and engagement, logistical and environmental barriers, concern about possible incarceration and lack of perceived benefit of treatment.

MOUD induction with buprenorphine has become the gold standard of treatment in OUD, secondary to its demonstrated abilities to reduce harm and save lives. Prehospital clinicians are the first contact health care professionals for many of these patients. In 2017, the United States Congress enacted the Protecting Patient Access to Emergency Medications Act (PPAEMA).

The PPAEMA permits EMS professionals to administer buprenorphine in the same manner it permits them to administer other controlled substances, such as fentanyl. Unlike most other controlled substances, however, EMS administration of buprenorphine for OUD requires that the authorizing medical director or other official have obtained an X-waiver because a standing order for buprenorphine technically qualifies as a prescription of the medication under federal law. Despite this law, buprenorphine use by EMS is relatively rare. The first case series published was out of Camden, New Jersey, where buprenorphine was given by EMS to three patients who had received Narcan for opioid overdose. Paramedics treated each patient with 16mg of buprenorphine to relieve and prevent withdrawal symptoms. Patients were provided with outpatient follow-up, irrespective of ED transport. There were no complications in giving prehospital buprenorphine.^6^

A second prehospital buprenorphine program was initiated in Contra Costa County, California. The program, known as the EMS Buprenorphine Use Pilot (EMSBUP) program,^7^ allowed responding paramedics to initiate MOUD with buprenorphine in the field for patients experiencing withdrawal symptoms independently or following naloxone reversal. In the first year of the study, 36 patients enrolled received prehospital buprenorphine. Of those patients receiving buprenorphine, only one patient signed out against medical advice on scene. All other patients were transported to an ED, and their clinical outcomes and seven-day and 30-day follow-ups were determined by the substance use navigator (SUN). Thirty-six of 36 patients had follow-up data obtained in the short term, and none experienced any precipitated withdrawal or other adverse outcomes. Patients had a 50% (18/36) rate of treatment retention at seven days, and 36% (14/36) were in treatment at 30 days.^7,8^

Another prehospital buprenorphine program was out of San Antonio, Texas. Over three years, a total of 263 patients were evaluated by EMS for MOUD induction, and 99 patients met the criteria and received at least one dose by EMS staff. There were no adverse events reported to either the EMS system or the receiving addiction treatment facility related to dosing by EMS clinicians in the prehospital environment. Specifically, no events of respiratory depression, subsequent overdose, or need for naloxone administration were observed.^9^

As the opioid addiction crisis continues unabated, community needs will remain substantial for the foreseeable future, potentially necessitating more frequent QRT intervention with consequent increases in programmatic service delivery, including MOUD. Despite the absence of patients who participated in this MOUD program, several successes were identified. The protocol for community-based care, which can increase patient access to care through the prehospital MOUD program, was established. Collaboration with community partners increases the available resources for patients struggling with OUD. The hospital’s expansion to prehospital MAT treatment provides another level of substance use disorder (SUD)/OUD treatment for patients.

The AFD was receptive to developing the prehospital MAT program. They initiated the training programs for their QRT members and participated in the state grant team meetings. Even though they could not enroll patients in the prehospital MOUD program, continued discussions about expanding the program to specific medic units in real-time (not just through the QRT) are ongoing.

Future studies may investigate engagement approaches, including timing, such as follow-up care and post-hospital discharge, as well as enhancing patient education efforts to address stigma and convey the benefits of MOUD more effectively. Finding the reasons for care refusal will help us address this treatment gap.

## Limitations

This study describes the process for instituting an EMS-based MAT program. The study’s major limitation was that it involved only one EMS system based in Akron, Ohio, and the program could not induct any prehospital patients with buprenorphine. A grant from the Ohio Department of Mental Health and Addiction Services supported the program. It required a collaborative approach with the city EMS and the two health systems that provided ED-based MAT. This may be difficult to implement in other systems, but the program has established a stepwise template for others.

## Conclusion

This prehospital MOUD program expanded the treatment options and opportunities available to those who have experienced an opioid overdose using EMS providers. Challenges were encountered in the expansion of services through this pathway. However, the program has succeeded in establishing new relationships, developing treatment protocols, designing and implementing team training, and identifying a new approach to prehospital MOUD treatment initiation that did not exist.
